# Cognitive decline in older adults in the UK during and after the COVID-19 pandemic: a longitudinal analysis of PROTECT study data

**DOI:** 10.1016/S2666-7568(23)00187-3

**Published:** 2023-11

**Authors:** Anne Corbett, Gareth Williams, Byron Creese, Adam Hampshire, Vincent Hayman, Abbie Palmer, Akos Filakovzsky, Kathryn Mills, Jeffrey Cummings, Dag Aarsland, Zunera Khan, Clive Ballard

**Affiliations:** University of Exeter Medical School, University of Exeter, Exeter, UK; Wolfson Centre for Age-Related Diseases, King’s College London, London, UK; University of Exeter Medical School, University of Exeter, Exeter, UK; Department of Brain Sciences, Faculty of Medicine, Imperial College London, London, UK; University of Exeter Medical School, University of Exeter, Exeter, UK; University of Exeter Medical School, University of Exeter, Exeter, UK; University of Exeter Medical School, University of Exeter, Exeter, UK; University of Exeter Medical School, University of Exeter, Exeter, UK; Chambers-Grundy Center for Transformative Neuroscience, Department of Brain Health, School of Integrated Health Sciences, University of Nevada, Las Vegas, Las Vegas, NV, USA; Institute of Psychiatry, Psychology and Neuroscience, King’s College London, London, UK; Institute of Psychiatry, Psychology and Neuroscience, King’s College London, London, UK; University of Exeter Medical School, University of Exeter, Exeter, UK

## Abstract

**Background:**

Although the long-term health effects of COVID-19 are increasingly recognised, the societal restrictions during the COVID-19 pandemic hold the potential for considerable detriment to cognitive and mental health, particularly because major dementia risk factors—such as those related to exercise and dietary habits—were affected during this period. We used longitudinal data from the PROTECT study to evaluate the effect of the pandemic on cognition in older adults in the UK.

**Methods:**

For this longitudinal analysis, we used computerised neuropsychology data from individuals aged 50 years and older participating in the PROTECT study in the UK. Data were collected from the same participants before the COVID-19 pandemic (March 1, 2019–Feb 29, 2020) and during its first (March 1, 2020–Feb 28, 2021) and second (March 1, 2021–Feb 28, 2022) years. We compared cognition across the three time periods using a linear mixed-effects model. Subgroup analyses were conducted in people with mild cognitive impairment and in people who reported a history of COVID-19, and an exploratory regression analysis identified factors associated with changes in cognitive trajectory.

**Findings:**

Pre-pandemic data were included for 3142 participants, of whom 1696 (54·0%) were women and 1446 (46·0%) were men, with a mean age of 67·5 years (SD 9·6, range 50–96). Significant worsening of executive function and working memory was observed in the first year of the pandemic across the whole cohort (effect size 0·15 [95% CI 0·12–0·17] for executive function and 0·51 [0·49–0·53] for working memory), in people with mild cognitive impairment (0·13 [0·07–0·20] and 0·40 [0·36–0·47]), and in people with a history of COVID-19 (0·24 [0·16–0·31] and 0·46 [0·39–0·53]). Worsening of working memory was sustained across the whole cohort in the second year of the pandemic (0·47; 0·44–0·49). Regression analysis indicated that cognitive decline was significantly associated with reduced exercise (p=0·0049; executive function) and increased alcohol use (p=0·049; working memory) across the whole cohort, as well as depression (p=0·011; working memory) in those with a history of COVID-19 and loneliness (p=0·0038; working memory) in those with mild cognitive impairment. In the second year of the pandemic, reduced exercise continued to affect executive function across the whole cohort, and associations were sustained between worsening working memory and increased alcohol use (p=0·0040), loneliness (p=0·042), and depression (p=0·014) in those with mild cognitive impairment, and reduced exercise (p=0·0029), loneliness (p=0·031) and depression (p=0·036) in those with a history of COVID-19.

**Interpretation:**

The COVID-19 pandemic resulted in a significant worsening of cognition in older adults, associated with changes in known dementia risk factors. The sustained decline in cognition highlights the need for public health interventions to mitigate the risk of dementia—particularly in people with mild cognitive impairment, in whom conversion to dementia within 5 years is a substantial risk. Long-term intervention for people with a history of COVID-19 should be considered to support cognitive health.

**Funding:**

National Institute for Health and Care Research.

## Introduction

The COVID-19 pandemic has had a far-reaching impact on society, health, and health-care systems. Most nations used strict social restrictions—including physical distancing, quarantine, and full societal lockdowns—that had not previously been experienced in living memory.^1^ The effects of these measures are yet to be fully established. Concerns were raised regarding the neuropsychological effects of the restrictions, which have particular relevance for older adults in the context of increased potential dementia risk.^2–4^ A 2020 *Lancet* Commission highlighted the major contributions of lifestyle and mental health factors to cognitive health, with modifiable risks contributing to 40% of dementia cases.^4^ These factors map closely to the population-wide changes in health and lifestyle seen during and after the lockdowns, raising the important question of the effect of the pandemic on cognitive health and risk across populations. In 2021, a systematic review^5^ covering 200 000 participants worldwide showed strong evidence of increased alcohol use during the pandemic, and another systematic review^6^ covering 86 000 participants found a reduction in physical activity and an increase in sedentary behaviour. Social restrictions also resulted in reduced social contact and networking.^7^ Social isolation is closely associated with loneliness, and these constructs contribute to depression.^8,9^ Large cohort studies have reported increasing prevalence of poor mental health indicators compared with pre-pandemic levels.^10–15^ Given the close relationship of these factors with dementia risk, the need to establish the effect of the pandemic on cognitive health, particularly in older adults, is urgent.

A link between COVID-19 and longer-term effects on cognition has been established, with cognitive deficits included as symptoms of post-COVID-19 condition.^16–24^ However, the effect of the pandemic on cognition more broadly is less clear. There is a considerable risk that the increased prevalence of mental ill health and changes to lifestyle could lead to accelerated cognitive decline.^3^ Effects on executive function would be of particular concern because this domain is most closely associated with daily functioning. A small evidence base indicates that older adults have experienced cognitive effects during the pandemic.^25^ Several preliminary studies report worsening cognition in people with mild cognitive impairment and dementia as a result of the pandemic and associated social restrictions.^26–28^ Individuals with preclinical cognitive deficits or those who were previously cognitively healthy could have been similarly likely to experience cognitive decline due to reduced social contact, physical exercise, and cognitive stimulation.^4^ Because of the impact of lockdowns on access to health care, these individuals are also likely to have missed opportunities for the early detection of cognitive change. As such, understanding the effect of the pandemic on cognitive health, what factors have driven any changes, and whether these changes are sustained beyond the lifting of restrictions, is crucial.

This study uses data from PROTECT, an online UK longitudinal ageing study, to investigate the effect of the COVID-19 pandemic on cognition in older adults (≥50 years). PROTECT is uniquely positioned because it holds data on cognition from 2015 onwards and collected continuous data throughout the pandemic through remote, computerised cognitive assessments. These assessments provide sensitivity to cognitive trajectory and preclinical change. Additional measures were introduced into the PROTECT assessment battery to capture variables unique to the pandemic situation. The resulting database of cognitive and health data, collected over 7 years, enables the analysis of longitudinal change and associated variables before, during, and after the pandemic.

## Methods

### Study design

We did a longitudinal analysis of data collected from the PROTECT study, which launched in the UK on Nov 3, 2015. PROTECT received ethical approval from the UK London Bridge National Research Ethics Committee (13/LO/1578). To ensure the analysis of consistent 12-month time periods, data collected between March 1, 2019 and Feb 28, 2022 were included in the main analysis. We labelled the timepoints as follows: 1 year pre-pandemic (March 1, 2019–Feb 29, 2020); pandemic year 1 (March 1, 2020–Feb 28, 2021, the period of intensive societal restrictions); and pandemic year 2 (March 1, 2021–Feb 28, 2022, the period of lifting of restrictions). The data analysed were from the same individuals at each timepoint.

Although the PROTECT study launched in 2015, recruitment was staggered, so the inclusion of data collected before 2019 would have compromised sample size and power. Nevertheless, assessments collected before March 1, 2019 were available for a smaller number of participants. These additional descriptive data were used to support further exploratory analyses to describe the cognitive trajectory over an additional 2-year period (March 1, 2017–Feb 28, 2019).

### Participants

At the time of data collection, participants in the PROTECT study were aged at least 50 years, were required to have access to a computer and the internet, and had not been diagnosed with dementia. Recruitment was completed via the study website following national publicity and signposting through partner cohorts and organisations. Participants provided electronic informed consent through the online registration process.

All participants provided demographic information at baseline through an online questionnaire adapted from the Office of National Statistics, which included age, gender, ethnicity, and education level. Education level was categorised from secondary education (GCSE or O Levels; score of 1) to doctorate (PhD; score of 6).

### Cognitive assessment

Participants in the PROTECT study complete computerised cognitive tests annually. These tests were the well validated Verbal Reasoning test, which tests logical reasoning and problem solving (executive function), and three working memory tests: the Paired Associate Learning test, the Digit Span test, and the Self-Ordered Search test, all described in detail in previous publications.^3^ These tests form part of a computerised neuropsychology battery known as Factors of Longitudinal Attention, Memory and Executive Function,^29^ which has validated sensitivity to detect cognitive change; changes measured by this battery were found to correlate significantly with changes in activities of daily living.^29^ For each individual task, the outcome measure is the total score of correct responses corrected for errors made. Scores from Paired Associate Learning, Self-Ordered Search, and Digit Span tasks can be combined to provide a validated composite measure for working memory.^29^ Participants take the cognitive tests up to three times over a period of 7 days at each annual timepoint as a single testing session.

### Mental health data collection

Participants complete annual questionnaires to provide self-reported, health-related data. Depression is assessed using the well validated, nine-item Patient Health Questionnaire (PHQ-9), which is widely used in the UK and is broadly equivalent to the 12-item General Health Questionnaire.^30^ PHQ-9 items are scored between 0 and 3, with total scores ranging from 0 to 27. Loneliness was assessed using two items from a broader mental health questionnaire that asked participants the questions “Do you often feel alone?” and “How often, in the past week, did you feel alone?”. These questions were scored almost never, sometimes, often, or almost always.

### Lifestyle data collection

We selected lifestyle factors that had the strongest evidence for being affected by the COVID-19 pandemic. Data were collected for each factor at each timepoint. Information on physical exercise was captured using a question about the regularity of exercise, to which participants could answer yes or no: “Have you done any physical activity lasting at least 20 minutes that has left you out of breath in the last month?”. Regularity of alcohol use was captured using the question “How many drinks containing alcohol do you have on a typical day when you are drinking?”, with possible answers 1 or 2; 3 or 4; 5 or 6; 7, 8, or 9; and 10 or more.

### Data analysis

Analysis was conducted across the whole cohort where data were available. The cohort size exceeds the required sample size of 2644, which provides a 99% confidence level that the real value is within 1% of the measured value for an analysis of this type.

For the primary analysis, cognition scores were assigned to the three pandemic year timepoints (1 year pre-pandemic, pandemic year 1, and pandemic year 2). A linear mixed-effects model was built with cognition score as the outcome, pandemic year as the explanatory variable, and individual specific random effects. As per standard practice, age as a continuous variable and sex were included as covariates as both of these factors have a well established relationship with cognition. The fitted model was used to calculate the differences between the estimated marginal means of the cognition scores corresponding to the pandemic year groups, including calculation of any difference in cognition in participants who did not complete either one or both of the pandemic year timepoints using Student’s *t* test. The R packages lmer and emmeans were used for the analysis. Results are reported as Cohen’s d effect sizes with corresponding 95% CIs and associated p values.

### Sensitivity analysis

As a sensitivity analysis, we used an alternative data analysis method. All data available for the cognitive tests for each participant were averaged over the three repetitions of the tests at each session to obtain a cognitive score for each cognitive test at each testing session. This method provides additional descriptive information, enabling us to report percentage change in cognitive function. We excluded participants with a current diagnosis of cancer (because intermittent treatment can affect cognition and the completion of cognitive tests) or Parkinson’s disease (because of the resulting motor impairment). Change was defined as the difference in cognitive score in each cognitive test from the start to end of each of the defined study year periods. Change in cognition in pandemic year 1 was compared with change in cognition in pandemic year 2, and a further analysis used the same approach to compare change in cognition in pandemic year 2 with change in cognition in the pre-pandemic year. To establish whether the cognitive changes during the time periods differed, we conducted ANCOVAs using the PROC MIXED method from SAS version 9.4 with age, gender, and education at baseline fitted as covariates, as these factors have established effects on cognitive performance. The analysis also controlled for the number of repetitions of the cognitive tests at each assessment timepoint for each participant.

### Exploratory analyses

In addition to the whole cohort, we also applied the main analysis method to two subgroups: participants with mild cognitive impairment, defined according to the National Institute for Aging published criteria (classified as 1·5 SD from the age-matched and gender-matched normative performance on at least one cognitive test at baseline); and participants who self-reported a history of COVID-19.

We conducted a further subanalysis on individual cohorts (the whole cohort, those with mild cognitive impairment, and those with a history of COVID-19) using hierarchical multivariable regression analyses in SAS version 9.4 to examine potential associations of altered cognition. The model included the following risk factors: loneliness (split into two groups: participants who answered almost never or sometimes to the relevant question and those who answered often or almost always), depression (participants with a PHQ-9 score ≥5), regularity of alcohol use (split into two groups: participants who answered <5 to the relevant question and those who answered ≥5), and physical exercise (split into two groups: participants responding yes or no to the relevant question). For the regression analysis, the three working memory cognitive tests were combined to provide a composite working memory factor score to limit the number of individual exploratory analyses.^29^ We calculated Cohen’s d effect sizes using the difference between the timepoint means and pooled mean, and used Cohen’s classification of effect sizes. Descriptive cognitive data from 2017 to 2022 were explored to compare the percentage change in cognitive function in previous years with the percentage change during the pandemic.

### Role of the funding source

The funder of the study had no role in study design, data collection, data analysis, data interpretation, or writing of the report.

## Results

Neuropsychological data were available for 3142 participants in the PROTECT study at the 1 year pre-pandemic timepoint, of whom 1696 (54·0%) were women and 1446 (46·0%) were men, with a mean age of 67·5 years (SD 9·6, range 50–96). 316 (10·1%) participants did not provide data for the pandemic year 1 timepoint and 428 (13·6%) did not provide data for the pandemic year 2 timepoint. No significant difference in pre-pandemic trajectories was found between the individuals who completed or did not complete either the year 1 (slope difference 0·02, *t* 0·70, p=0·48) or year 2 (−0·02, −0·61, p=0·54) assessments. Reasons for not completing the cognitive tests were unknown. 752 (23·9%) of 3142 participants reported having COVID-19 during the period March 1, 2020–Feb 28, 2022. 147 (4·7 %) of 3142 participants fulfilled criteria for mild cognitive impairment (table 1).

**Figure 1:**
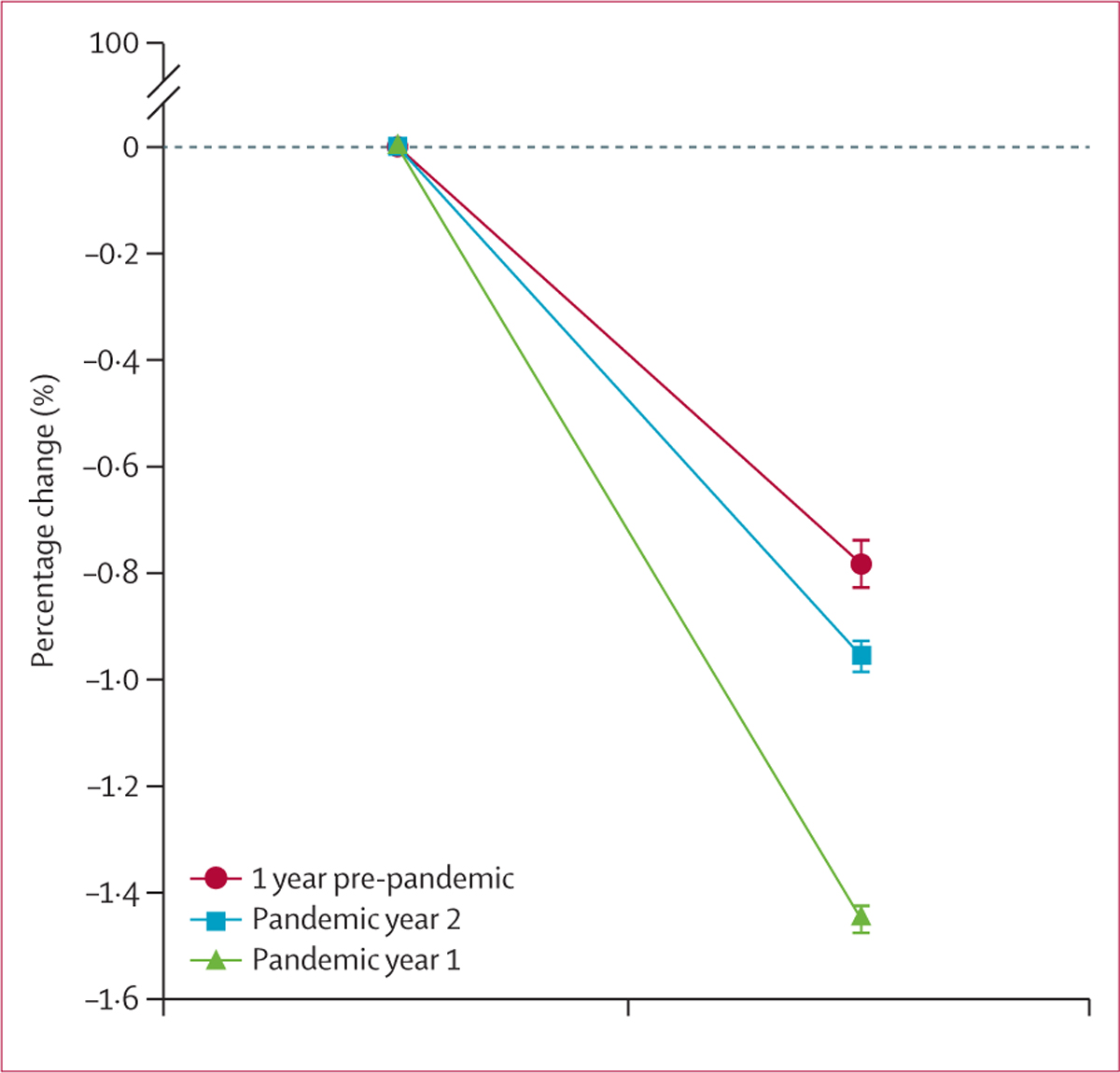
Percentage change in executive function cognitive test score for the whole cohort Data are mean (SE).

**Figure 2:**
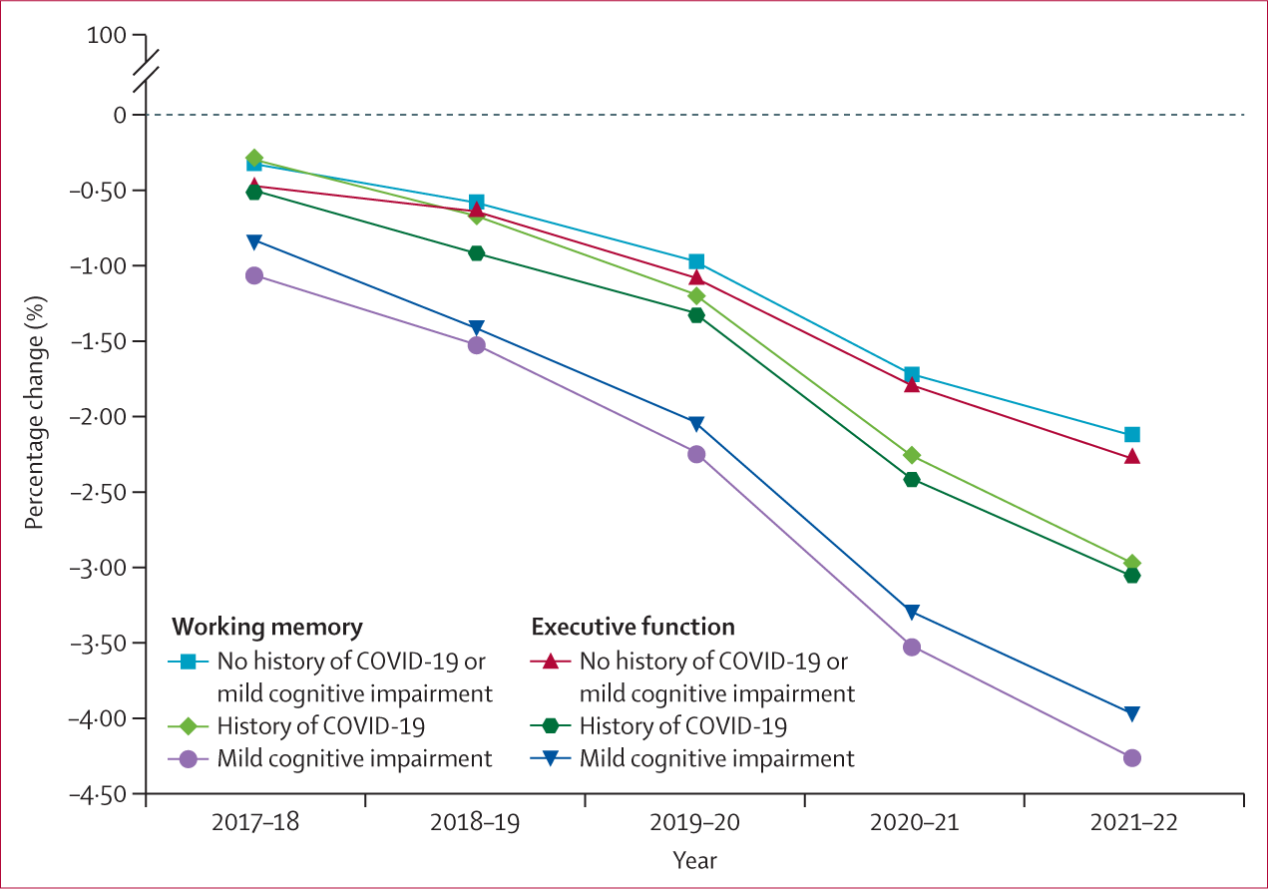
Percentage change in executive function and working memory in the whole cohort and in subgroups of participants with mild cognitive impairment and a history of COVID-19

Analysis of cognitive performance showed significant worsening of executive function and working memory trajectory during the first year of the COVID-19 pandemic compared with the pre-pandemic year (figure 1, table 2). This effect was sustained for working memory into the second year of the pandemic, with a continuation of accelerated decline relative to pre-pandemic levels (table 2). As a sensitivity analysis, we repeated this analysis using a dataset that excluded people with mild cognitive impairment or a history of COVID-19. Significant differences between the pre-pandemic year and pandemic year 1 were still evident for executive function (effect size 0·15; 95% CI 0·13–0.17; p<0·0001) and working memory (0·53; 0·50–0·53; p<0·0001). A further sensitivity analysis, using average scores analysed by ANCOVA, also showed a worsening in the trajectory of both executive function and working memory in both pandemic year 1 and pandemic year 2 compared to the pre-pandemic year (figure 2, appendix).

In people with mild cognitive impairment, a significant, sustained worsening of executive function was seen during the first and second years of the pandemic compared with the pre-pandemic year. Significant worsening of working memory was also observed across both pandemic years compared with the pre-pandemic period (table 2).

In people who reported having COVID-19 at some point between March 1, 2020 and Feb 28, 2022, a significant worsening of executive function and working memory was observed in the first year of the pandemic compared with the pre-pandemic year, an effect that was sustained in working memory in the second year of the pandemic (table 2).

All groups had a greater rate of change in cognition during the pandemic than in the pre-pandemic year. In the whole cohort, executive function declined by a mean of 0·61% and working memory by a mean of 0·64% in the year before the pandemic; however, across both years of the pandemic these declines accelerated to 1·24% (49% greater decline overall) for executive function and 1·16% (55% greater decline overall) for working memory. An increased rate of decline during the pandemic was also seen in the subgroup of participants with a history of COVID-19, both in executive function (0·81% decline pre-pandemic to 1·75% decline during the pandemic; 46% greater decline overall) and working memory (0·89% to 1·78%; 50% greater), and in the subgroup of participants with mild cognitive impairment, both in executive function (1·2% to 1·94%; 62% greater) and working memory (1·17% to 2·03%; 58% greater; figure 2).

We conducted an exploratory regression analysis to evaluate the associations between accelerated cognitive decline and known dementia risk factors. The aim of this analysis was to identify potential associations that can be explored further in future studies to establish causality and size of effect. In the first year of the pandemic, significant associations were seen between cognitive decline and both decreased frequency of exercise and increased alcohol use—these effects were seen both across the overall cohort and in the subgroups of people with mild cognitive impairment and those with a history of COVID-19. In people with mild cognitive impairment, loneliness (working memory β −1·648, 95% CI −2·835 to −0·461; p=0·0038) and depression (working memory β −1·688, −2·861 to −0·515; p=0·051) were associated with cognitive decline, and in people with a history of COVID-19, an association was observed between depression and cognitive decline (working memory β −2·279, −4·489 to −0·069; p=0·011; executive function β −4·913, −9·655 to −0·171; p=0·054; table 3) in the first year of the pandemic.

In the second year of the pandemic, decreased frequency of exercise was the only factor that continued to affect executive function across the whole cohort. However, in people with mild cognitive impairment, increased alcohol use (β −2·331, 95% CI −3·952 to −0·710; p=0·0040), loneliness (β −1·366, −2·183 to −0·549; p=0·042), and depression (β −1·540, −2·941 to −0·139, p=0·014) continued to be associated with worsening working memory, and in people with a history of COVID-19, associations were still evident between working memory and decreased frequency of exercise (β −1·969, −3·470 to −0·468; p=0·0029), loneliness (β −3·033, −5·672 to −0·394; p=0·031), and depression (β −3·878, −7·662 to −0·094; p=0·036; table 3).

## Discussion

We found that people aged 50 years and older in the UK had accelerated decline in executive function and working memory during the first year of the COVID-19 pandemic, during which the UK was subjected to three societal lockdowns for a total period of 6 months. Notably, however, this worsening in working memory persisted in the second year of the pandemic, after the social restrictions had eased. The scale of change is also of note, with all groups—the whole cohort and the individual subgroups—showing more than a 50% greater decline in working memory and executive function and many effect sizes reaching a clinically significant threshold of greater than 0·3. The subgroup analyses indicated the same effect, with more rapid cognitive decline in both groups than in the overall cohort. The mild cognitive impairment subgroup represents individuals at the highest risk of dementia, with a conversion rate of 10% each year.^31^ The data indicate that the pandemic conditions have accelerated cognitive decline in these individuals, and a key emerging question is whether their risk of conversion to dementia has also increased. The worsening of cognition in people with a history of COVID-19 aligns with literature reports of the cognitive effects of the disease, in which up to 78% of people report cognitive impairment.^16^ An important question is whether the pattern of significant differences in cognitive decline are meaningful. Clinical meaningfulness can be evaluated in several ways—for example, based on effect size (>0·3 is often taken as an appropriate threshold) or consistency between outcomes. With respect to the difference in cognitive decline between the first year of the pandemic and the pre-pandemic year, the primary outcome (executive function) exceeded this effect size threshold, and several further analyses exceeded the threshold in the subgroup analyses. In addition, although the effect sizes were smaller in several of the other secondary analyses, we observed consistent, significant changes across different tests. On this basis, there is clear justification to support an interpretation of the change as clinically meaningful.

The regression analysis, which was conducted as an exploratory analysis, further adds to our understanding of the factors that could be associated with accelerated cognitive decline. During the first year of the pandemic, reduced exercise and increased alcohol intake were significantly associated with the worsening of cognitive trajectory. Exercise levels are well established risk factors for cognitive decline. For most adults, the pandemic conditions disrupted routines and led to reduced regularity, intensity, and duration of exercise.^32^ There is clear evidence that alcohol use increased during the pandemic, with more than one in six UK adults increasing their alcohol consumption.^13^ We therefore hypothesise that reduced exercise and increased alcohol consumption could have affected cognition. However, we cannot assume causality from this study, and this hypothesis would need further investigation.

Depression and loneliness were also associated with some aspects of accelerated cognitive decline in the subgroups of people with mild cognitive impairment and those who reported a history of COVID-19. Depression is a known risk factor for cognitive decline, and the lockdown conditions have been associated with worsening of depressive symptoms.^25^ People reporting high levels of loneliness also showed increased cognitive decline, reflecting the known effect of social isolation and loss of social contact on reduced cognitive health. Further work is needed to clarify the relationship between depression, loneliness, and cognitive decline, particularly in people with early cognitive impairment.

In the exploratory regression analysis examining cognitive decline over the second year of the pandemic, exercise was again associated with a decline in executive function. Ongoing concerns about the pandemic and a shift to more virtual communication forms, leading to less time spent out of the house and a less active lifestyle, could explain this continued association beyond the immediate lockdown periods; however, this hypothesis requires further examination. In people with mild cognitive impairment, alcohol use, loneliness, and depression were significantly associated with decline in executive function. In the context of the existing evidence base regarding the risk of dementia in people with depression, the effect of depression on cognitive decline in people with mild cognitive impairment is an important area for future consideration. In people with a history of COVID-19, exercise, depression, and loneliness were all associated with some aspects of accelerated cognitive decline. These findings emphasise the long-term cognitive risk in this newly defined patient group, and highlight the need to consider whether lifestyle and mental health interventions could benefit cognitive health in people who have had COVID-19. Given the scale of the pandemic, a major initiative will be required if we are to avoid serious public health effects in the medium-to-long term.

This study provides insight into the effect of the COVID-19 pandemic on cognitive health in older adults. The PROTECT study is in a strong position to explore this question, owing to its longitudinal dataset with a consistent cohort of participants, the large cohort size, and remote-testing infrastructure that enabled continuous data capture before, during, and after the pandemic, unlike most published analyses. To our knowledge, this study provides the largest-scale analysis of longitudinal cognitive data collected during the COVID-19 pandemic, using sensitive computerised cognitive tests to detect domain-specific changes alongside extensive health data. We also acknowledge some important limitations. The health data are self-reported and so are subject to a degree of uncertainty, although it is increasingly acknowledged that this method of data collection often delivers more accurate, honest responses than in-person questionnaires.^33^ The PROTECT cohort is self-selected and has a current bias towards particular demographic groups, especially individuals with higher levels of education; this bias is important to consider when interpreting the results, and could mean that the outcomes are not representative of trends in the overall population. The subgroup analyses were exploratory and so should be interpreted with caution, and the number of participants in the mild cognitive impairment group was relatively small. In addition, although the analysis explored data regarding known risk factors and cognitive decline, causality cannot be assumed from these findings. Finally, other confounding factors that were not included in this analysis might be present.

These findings highlight a pattern of associations between exercise, alcohol use, depression, and loneliness—all of which are known risk factors for dementia—and cognitive decline during the COVID-19 pandemic. Although direct causality cannot be assumed from these data, the increase in depression, reduction in regularity of exercise, and increase in alcohol use across the population during the pandemic is well known. As such, there is a clear need to address these changes in lifestyle behaviour as a public health priority, and on the basis of the patterns of associations seen in the current study, we would hypothesise that interventions targeting these behaviours could benefit cognition.

## Supplementary Material

Supplementary Appendix

## Figures and Tables

**Table 1:** Baseline characteristics

	Whole cohort (n=3142)	Mild cognitive impairment (n=147)	History of COVID-19 (n=752)

Age, years	67·5 (9·6, 50–96)	68·7 (7·5, 58–96)	67·1 (8·4, 50–95)
Education level	4 (1–4)	3 (1—4)	4 (1–4)
Gender			
Women	1696 (54·0%)	94 (63·9%)	459 (61·0%)
Men	1446 (46·0%)	53 (36·1%)	293 (39·0%)
Ethnicity			
White	3091 (98·4%)	143 (97·3%)	739 (98·3%)
Mixed or multiple background	19 (0·6%)	1 (0·7%)	4 (0·5%)
Asian or Asian British	22 (0·7%)	1 (0·7%)	7 (0·9%)
Black, African, or Caribbean	4 (0·1%)	1 (0·7%)	0
Other	6 (0·2%)	1 (0·7%)	2 (0.3%)

Data are mean (SD, range), median (IQR), or n (%).

**Table 2:** Cognition during years 1 and 2 of the COVID-19 pandemic, compared with the pre-pandemic year, in the whole cohort and two subgroups

	Pandemic year 1		Pandemic year 2	
	Cohen’s d effect size (95% CI)	p value	Cohen’s d effect size (95% CI)	p value

**Whole cohort**				
Executive function				
Verbal Reasoning	0·15 (0·12 to 0·17)	<0·0001	0·02 (0·00 to 0·04)	0·12
Working memory				
Paired Associate Learning	0·77 (0·30 to 0·37)	<0·0001	0·74 (0·72 to 0·76)	<0·0001
Self-Ordered Search	0·15 (0·13 to 0·18)	<0·0001	0·15 (0·13 to 0·18)	<0·0001
Digit Span	0·19 (0·17 to 0·21)	<0·0001	0·14 (0·12 to 0·16)	<0·0001
Composite	0·51 (0·49 to 0·53)	<0·0001	0·47 (0·44 to 0·49)	<0·0001
**Mild cognitive impairment**				
Executive function				
Verbal Reasoning	0·13 (0·07 to 0·20)	<0·0001	0·08 (−0·04 to 0·08)	0·038
Working memory				
Paired Associate Learning	0·72 (0·66 to 0·78)	<0·0001	0·66 (0·60 to 0·71)	<0·0001
Self-Ordered Search	0·02 (−0·05 to 0·08)	0·86	0·02 (−0·04 to 0·08)	0·82
Digit Span	0·27 (0·20 to 0·33)	<0·0001	0·16 (0·09 to 0·22)	<0·0001
Composite	0·40 (0·36 to 0·47)	<0·0001	0·32 (0·26 to 0·39)	<0·0001
**History of COVID-19**				
Executive function				
Verbal Reasoning	0·24 (0·16 to 0·31)	<0·0001	0·01 (−0·05 to 0·08)	0·12
Working memory				
Paired Associate Learning	0·75 (0·68 to 0·82)	<0·0001	0·73 (0·66 to 0·79)	<0·0001
Self-Ordered Search	0·11 (0·04 to 0·18)	0·0069	0·15 (0·08 to 0·21)	<0·0001
Digit Span	0·12 (0·05 to 0·19)	0·0018	0·10 (0·03 to 0·16)	0·0085
Composite	0·46 (0·39 to 0·53)	<0·0001	0·46 (0·39 to 0·52)	<0·0001

**Table 3:** Association of lifestyle, social, and mental health factors with worsening of cognitive trajectory across the 2 years of the COVID-19 pandemic in the whole cohort and two subgroups

	Working memory (mean of Paired Associate Learning, Self-Ordered Search, and Digit Span tasks)		Executive function (Verbal Reasoning test)	
	β (95% CI, SE)	p value	β (95% CI, SE)	p value

**Pandemic year 1**				
Whole cohort				
Exercise	−3·344 (−6·402 to −0·286; 0·146)	0·61	−1·694 (−2·778 to −0·610; 0·311)	0·0049
Alcohol	−1·333 (−2·327 to −0·339; 0·173)	0·049	−1·675 (−2·760 to −0·590; 0·301)	0·92
Loneliness	−0·348 (−0·439 to −0·257; 0·131)	0·061	−1·813 (−3·156 to −0·470; 0·240)	0·84
Depression	−3·112 (−6·161 to −0·063; 0·032)	0·51	−1·163 (−2·155 to −0·171; 0·087)	0·073
History of COVID-19				
Exercise	−2·263 (−4·181 to −0·345; 0·176)	0·014	−3·354 (−6·173 to −0·535; 0·273)	0·0020
Alcohol	−1·050 (−2·082 to −0·018; 0·009)	0·023	−1·661 (−2·914 to −0·408; 0·208)	0·11
Loneliness	−3·438 (−6·590 to −0·286; 0·146)	0·072	−4·038 (−7·555 to −0·521; 0·266)	0·092
Depression	−2·279 (−4·489 to −0·069; 0·035)	0·011	−4·913 (−9·655 to −0·171; 0·087)	0·054
Mild cognitive impairment				
Exercise	−2·741 (−4·870 to −0·612; 0·312)	0·0051	−1·509 (−2·604 to −0·414; 0·211)	0·78
Alcohol	−2·281 (−4·362 to −0·200; 0·102)	0·031	−2·772 (−4·766 to −0·778; 0·397)	0·038
Loneliness	−1·648 (−2·835 to −0·461; 0·235)	0·0038	−1·591 (−2·708 to −0·474; 0·242)	0·53
Depression	−1·688 (−2·861 to −0·515; 0·263)	0·051	−1·732 (−3·292 to −0·172; 0·088)	0·28
**Pandemic year 2**				
Whole cohort				
Exercise	−2·379 (−4·203 to −0·555; 0·283)	0·019	1·194 (−0·559 to 2·947; 0·285)	0·062
Alcohol	−0·259 (−0·022 to −0·496; 0·253)	0·21	−0·198 (−0·417 to 0·021; 0·213)	0·083
Loneliness	−0·468 (−0·381 to −0·555; 0·283)	0·92	−1·253 (−1·951 to −0·555; 0·283)	0·0060
Depression	−2·150 (−4·267to −0·033; 0·017)	0·86	−1·027 (−1·915 to −0·139; 0·071)	0·93
History of COVID-19				
Exercise	−1·969 (−3·470 to −0·468; 0·239)	0·0029	−3·014 (−5·618 to −0·410; 0·209)	0·14
Alcohol	1·402 (−0·500 to 3·304; 0·255)	0·24	−1·107 (−1·659 to −0·555; 0·283)	0·30
Loneliness	−3·033 (−5·672 to −0·394; 0·201)	0·031	−3·115 (−5·695 to −0·535; 0·273)	0·051
Depression	−3·878 (−7·662 to −0·094; 0·048)	0·036	−3·097 (−6·141 to −0·053; 0·027)	0·89
Mild cognitive impairment				
Exercise	−1·738 (−2·998 to −0·478; 0·244)	0·44	−0·904 (−1·320 to −0·488; 0·249)	0·041
Alcohol	−2·331 (−3·952 to −0·710; 0·362)	0·0040	−1·650 (−2·808 to −0·492; 0·251)	0·93
Loneliness	−1·366 (−2·183 to −0·549; 0·280)	0·042	−0·210 (−0·525 to 0·105; 0·268)	0·062
Depression	−1·540 (−2·941 to −0·139; 0·071)	0·014	−0·390 (−0·660 to −0·120; 0·061)	0·42

## Data Availability

Individual de-identified participant data that underlie this reported study are available as per the PROTECT study protocol up to 10 years after the study end date. Investigators wishing to access the data require approval through the PROTECT study committee, which can be sought by applying through the PROTECT study with a full analysis proposal. Investigators will need to sign a data access agreement. Approved requests will be able to access data from a secure web link for up to 5 years subject to approval. For further information, contact protect.data@exeter.ac.uk.

## Research in context

### Evidence before this study

We searched PubMed for studies conducted between Jan 1, 2020, and Sept 30, 2022, using the search terms ([“Cognition” OR “cognitive” OR “dementia”] AND [“COVID” OR “COVID-19”] AND [“pandemic” OR “Lockdown”] AND “longitudinal” OR “cohort”). The date range was dictated by the timeline of the COVID-19 pandemic. Studies of cognitive outcomes were very small in scale, had a cross-sectional design, or had longitudinal data limited to the pandemic timescale and no comparisons with pre-pandemic rates of decline. Some longitudinal studies of lifestyle and medical factors had longer timescales, showing the effects of the pandemic on the worsening of depression and social isolation and on physical activity and alcohol use. Several systematic reviews and meta-analyses have been published on the effects of the pandemic, concluding a widespread detriment to mental health and wellbeing.

### Added value of this study

This study provides large-scale longitudinal data—including pre-pandemic data from the same individuals—to enable evaluation of the effect of the COVID-19 pandemic period on the cognitive trajectory of older adults. We report domain-specific changes in cognition and evaluate effects in groups of individuals at high risk of cognitive decline—those with mild cognitive impairment and those with a history of COVID-19. This study provides insight into cognitive health throughout the pandemic in the context of pre-pandemic cognition, and highlights key drivers of change that might be sensitive to targeted interventions and clinical support.

### Implications of all the available evidence

The sustained worsening of cognitive trajectory in older adults during the COVID-19 pandemic in comparison with the pre-pandemic period has major implications for public health and dementia risk, and emphasises the need to incorporate measures to support cognitive health as part of planning for further COVID-19 outbreaks and future pandemics. In particular, the evidence highlights the need to better support groups who are already at risk of cognitive decline and dementia. This need is particularly immediate for people with mild cognitive impairment, in whom the cognitive effects of the pandemic could have a direct effect on conversion to dementia over the next 5 years.
