# Supplementary material for: Cognitive decline in older adults in the UK during and after the COVID-19 pandemic: a longitudinal analysis of PROTECT study data
Source: Lancet Healthy Longev. Author manuscript; Available in PMC 2024 Nov 1. (PMC10720396; doi:10.1016/S2666-7568(23)00187-3)
Supplement: Supplementary Appendix [file NIHMS1943871-supplement-Supplementary_Appendix.pdf]

THE LANCET
Healthy Longevity

Supplementary appendix

This appendix formed part of the original submission and has been peer reviewed. We post it as supplied by the authors.

Supplement to: Corbett A, Williams G, Creese B, et al. Cognitive decline in older adults in the UK during and after the COVID-19 pandemic: a longitudinal analysis of PROTECT study data. *Lancet Healthy Longev* 2023; **4:** e591–99.

Supplementary Table 1: Sensitivity Analysis: Cognitive trajectories in the whole cohort across two cognitive domains, comparing the first and second year of the pandemic respectively with change in the pre-pandemic year

Domain	Cognitive Test	Year 1 of Pandemic			Year 2 of Pandemic		
		F	P	Cohen's d	F	P	Cohen's d
Executive Function	Verbal Reasoning	31.42	0.0006	0.61	32.61	0.043	0.19
Working Memory	Paired-Associate Learning	66.09	0.040	0.16	73.41	0.059	0.08
	Self-Ordered Search	31.72	0.022	0.19	44.62	0.002	0.27
	Digit- Span	38.46	0.04	0.21	39.83	0.0008	0.11
